# Knee angle reproduction tests: influences of body orientation, movement direction and limb dominance

**DOI:** 10.1055/a-2526-9372

**Published:** 2025-02-20

**Authors:** Juliane Wieber, Abigail Preece, Robert Rein, Bjoern Braunstein

**Affiliations:** 1 Exercise Physiology and Sports Medicine, Olympic Training Centre Berlin, Berlin, Germany; 2 14915 Sports and Exercise Medicine, University of Hamburg Faculty of Education Psychology and Physical Science, Hamburg, Germany; 3 14926 Institute of Movement and Neuroscience, German Sport University Cologne, Cologne, Germany; 4 14926 Institute of Exercise Training and Sport Informatics, German Sport University Cologne, Cologne, Germany; 5 14926 Institute of Biomechanics and Orthopaedics, German Sport University Cologne, Cologne, Germany; 6 14926 German Research Centre of Elite Sport, German Sport University Cologne, Cologne, Germany; 7 14926 Centre for Health and Integrative Physiology in Space, German Sport University Cologne, Cologne, Germany

**Keywords:** return to sport, return to competition, physiotherapy, orthopaedics, proprioception, joint position sense

## Abstract

Applying joint position sense tests under different test conditions may introduce reproduction error bias, which can result in different therapeutic consequences. This study investigated the effects of body orientation, movement direction, and limb dominance on the active knee angle reproduction error. Subjects underwent active contralateral knee angle reproduction tests in a seated versus prone position, from a starting point of knee flexion versus knee extension, and with the dominant versus nondominant limb setting the target angle. The test order was randomly determined for each subject. The primary outcome was the absolute active knee angle reproduction error (°). The data of 54 healthy subjects (mean±standard deviation, age: 26±5 years, height: 174±11 cm, body mass: 69.9±14.4 kg, and Tegner activity score: 5.8±1.9) showed that the reproduction error was greater in the seated position than in the prone position. The use of the dominant limb as the reference limb was associated with significantly greater errors in the seated position, but not in the prone position. In conclusion, directly comparing the results obtained in the prone and seated positions is not recommended. However, the dominance of the reference limb might be relevant when testing patients and comparing healthy and injured knees.

## Abbreviations

**AAE**: Absolute angular error
**ANOVA**: Analysis of variance
**CI**: Confidence interval
**JPS**: Joint position sense
**LMM**: Linear mixed model

## Introduction

Proprioception is defined as the perception of the body’s position and movement and is described as a complex construct that has not yet been fully examined 1 2 . The outcomes of studies on proprioception play an important role in injury prevention and during the process of rehabilitation 3 4 . Impaired proprioception is accompanied by a reduction in coordination ability, which is a possible reason for knee injuries, especially anterior cruciate ligament ruptures in pivoting sports 3 .

Assessing the joint position sense (JPS) is the most widely used method for measuring the knee’s proprioceptive capability, and JPS tests are performed as a part of almost every orthopedic rehabilitation program 5 6 . The accuracy of such tests is of utmost clinical importance as the results are used to justify exercise programs and to evaluate the success of treatment programs 6 . However, the protocols used to test the knee’s proprioceptive capability vary, which leads to different test results 1 7 . Elangovan et al. suggested that differences in test protocols do not denote methodological weaknesses but are due to various physiological factors that vary across protocols and thus influence JPS results 8 .

A combination of different sensory inputs for detecting, generating, and stimulating the joint position, movement velocity, direction, and force forms the basis of proprioception 2 . There are various types of sensory mechanoreceptors located in joint tissues, muscles, ligaments, tendons, and the skin 8 . These receptors are a part of the vestibular, visual, and somatosensory systems and are responsible for recognizing the body’s position and the orientation of body segments with respect to other body segments 6 8 9 . It has been shown that, when individuals actively reproduce different knee joint angles, there are significant differences in the triggered mechanoreceptors, depending primarily on the position and direction of the limb movement 5 7 10 11 12 13 14 15 16 17 . The direction of movement has been shown to influence the knee’s JPS: higher average error values are recorded when the leg moves from extension to flexion 7 14 15 18 19 . Furthermore, the body’s position has been shown to influence the knee angle reproduction error, with greater deviations recorded in the prone position 7 16 20 . However, there is conflicting evidence that limb dominance is associated with proprioceptive acuity; it has been shown to be an influencing factor in some studies 15 21 22 23 24 but not in other studies 23 25 . To progress the clinical implementation of a standardized JPS test, the aim of this study was to investigate the effects of body orientation, movement direction, and limb dominance on knee angle reproduction errors measured in an active contralateral knee angle reproduction test. It was hypothesized reproduction errors to deviate under varying test conditions.

## Materials and Methods

An observational clinical trial with blinded outcome assessors was performed in January 2024. The subject recruitment extended from October to December 2023. Due to the study’s design, no follow-up was required. The study was conducted in accordance with the Declaration of Helsinki of 1964 and its later amendments or comparable ethical standards and was approved by the Institutional Ethics Boards of the German Sport University Cologne and the University of Hamburg. The experimental design was preregistered on the Open Science Framework (DOI 10.17605/OSF.IO/AFWRP ) and adhered to the Strengthening the Reporting of Observational Studies in Epidemiology guidelines 26 . The subjects were recruited through voluntary participation. Computer-generated tables were used to determine the order in which the subjects were exposed to the following test conditions: extension versus flexion (direction), sitting versus laying (position), and the dominant leg adjusting to the target angle versus the dominant leg setting the reference angle (simulation). All standardized assessments were performed by a graduated exercise/sport scientist. All data were collected at the German Sport University Cologne. To determine the required sample size, the statistical power was set at 0.80 and the effect size was set at *d* =0.60, based on previous research 7 8 20 27 28 . A priori power analysis resulted in a required sample size of at least 20 subjects (G*Power, Version 3.1.9.4). The exclusion criteria were physical limitations, such as a general neuronal disease or a history of muscles, ligaments, tendons, or bone injury in the lower extremities. Furthermore, the subjects were not allowed to have chronical diseases which could lead to sensory disfunctions. Each subject provided a written informed consent for the participation, data collection, and image publication prior to enrolment.

### JPS test

Prior to testing, the subjects were familiarized with the study protocol via standardized verbal instructions. The active contralateral knee angle reproduction test was performed over five trials in two different positions: sitting and prone. The subjects were blindfolded to prevent the visual feedback bias. Each lower leg was passively moved from a starting point of knee flexion (starting angle of 90°) or knee extension (starting angle of 0°) to a random target angle between 50° and 70°. To minimize detection and performance bias, the subjects were retrospectively asked about their dominant side. Under all conditions, the subject’s knee joint was passively moved at a slow speed by the examiner. Once the examiner had brought the knee to the target angle, the subject had to hold the knee position without further assistance of the examiner. The subject was asked to actively reproduce the target angle with the contralateral leg while the other leg remains in the reference position. The subject stopped moving the leg when they perceived that the target angle had been replicated and gave a verbal signal. The knees were maintained at the final angles for approximately 3 s. Subjects were not permitted to adjust the angle of the contralateral leg after they signaled that they had replicated the target angle. During the test, each subject laid or sat on an adjustable physiotherapy bench. In the seated position, approximately 5 cm of an overhang of the leg was instigated to minimize cutaneous cues. Testing was conducted in a quiet and isolated room, and the subjects wore loose-fitting shorts to minimize external stimulation. To minimize proprioceptive transfer between consecutive tests, subjects walked around for approximately 2 min between tests. The lateral knee joint cavity on both legs was marked at the beginning of the test session and remained unchanged between trials. An electrical goniometer (Vernier Software & Technology, Dynatech) was attached to the lower limb, and a fulcrum was aligned with the lateral knee joint line. One arm of the goniometer was positioned parallel to the line joining the greater trochanter and the fulcrum, while the other arm was positioned along the line joining the fulcrum and the lateral malleolus. The reliability of comparable active contralateral test procedures was found to be fair to good for the prone (intraclass correlation coefficient [ICC]: 0.50–0.68) and sitting positions (ICC: 0.26–0.65) 16 . Inter-rater and intra-rater reliabilities using a long arm goniometer were found to be high (ICC: > 0.98) 29 .

### Outcome measures

The outcome measure was the absolute angular error (AAE), measured in degrees (°). To calculate the mean AAE, the absolute differences between the target angles and the actual produced angles were calculated.

### Statistical analysis

Statistical analyses were performed using R (Version 4.3.2). Statisticians were blinded to the different test procedures. Data were checked for missing values, distributions, and outliers and descriptively summarized as means±standard deviations (SDs), standard errors (SEs), and 95% confidence intervals (CIs). Statistical outliers were calculated by *Z* -scores and excluded if ≥1.96 SD 30 . Homogeneity of variance was tested using the Levene test. To account for the repeated measures and potential subject-specific variations, a linear mixed model (LMM) was employed. The fixed effects included the body orientation, the direction of movement, and limb dominance and the individual subjects were modelled as random effects. An analysis of variance (ANOVA) was conducted using Satterthwaite’s method to examine the overall differences and interaction effects among the body orientation, the direction of movement, and limb dominance. The dependent variable was the AAE and the independent variables were the body orientation, the direction of movement and limb dominance. When the overall ANOVA indicated significance, post hoc Tukey’s HSD tests were applied to further explore pairwise differences. Any missing data were addressed using listwise deletion. *P* -values less than 0.05 were considered to indicate a statistical significance ( *α* =0.05). The effect size was calculated and interpreted as Cohen’s *d* for small (0.20), medium (0.50), or large (0.80) effects.

## Results

### Descriptive statistics

The study included 54 healthy subjects (mean±SD, age: 26±5 years, height: 174±11 cm, body mass: 69.9±14.4 kg, and Tegner activity score: 5.8±1.9; 20 males and 34 females). There were no dropouts. The AAE values associated with the different body positions, directions of movement, and uses of the dominant leg are shown in Table 1 .

**Table:** **Table 1** Absolute reproduction errors recorded in a knee angle reproduction test in which specific conditions were varied ( *n* =54).

Position	Type	Direction	Mean (°)	SD (°)	Min (°)	Max (°)
Prone	dom_adj	Ex	5.52	3.80	0.01	18.64
Prone	dom_adj	Flex	5.26	4.61	0.10	27.78
Prone	dom_ref	Ex	6.05	3.83	0.09	17.71
Prone	dom_ref	Flex	4.52	3.39	0.00	14.26
Sitting	dom_adj	Ex	7.78	5.70	0.03	24.87
Sitting	dom_adj	Flex	7.05	5.07	0.09	20.57
Sitting	dom_ref	Ex	8.89	6.90	0.03	31.59
Sitting	dom_ref	Flex	8.05	5.91	0.20	26.07

Abbreviations: dom_adj, the dominant leg was adjusted to replicate the target angle; dom_ref, the dominant leg was set at the reference angle; ex, extension; flex, flexion; Max, the highest absolute angular error; Min, the lowest absolute angular error.

### Influences of the body orientation, the direction of movement and limb dominance on the resulting angular error

**Fig. 1:**
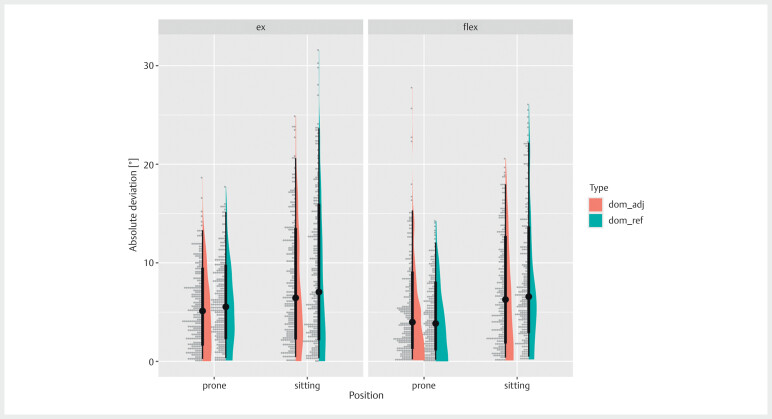
Boxplot of the difference in the knee angle reproduction error between the prone and seated positions: dom_adj, the dominant leg was adjusted to replicate the target angle; dom_ref, the dominant leg was set at the reference angle.

Investigation of the residual plots from the LMM analysis did not indicate any relevant deviations from the testing assumption, suggesting homoscedasticity. However, posterior predictive checks indicated that the simulated and observed data differed. To improve the model fit, the AAE values were root-mean square transformed. Subsequent model checking indicated an improvement in the model fit, but did not alter the interpretation of the analysis results. Thus, further analysis was performed using the raw AAE values. However, to guard against erroneous interpretations of the statistical significance, the *α* -level was lowered to 0.01. The results of the ANOVA based on the LMM model fit indicated several statistically significant effects. The main effects for the body orientation ( *F* (1, 54)=23.53, *p* < 0.001) and limb dominance ( *F* (1, 1977)=8.44, *p* =0.004) were statistically significant, whereas for the direction of movement ( *F* (1, 54)=6.28, *p* =0.015) no statistically significant effect was found. The interaction effects for the body position by limb dominance ( *F* (1, 1977)=12.80, *p* < 0.001) was statistically significant. The movement direction by the body position ( *F* (1, 54)=0.02, *p* =0.875) and the movement direction by limb dominance ( *F* (1, 1977)=4.34, *p* =0.036) were not found to be statistically significant ( Fig. 1 ).

### Differences between the dominant and nondominant limbs

**Fig. 2:**
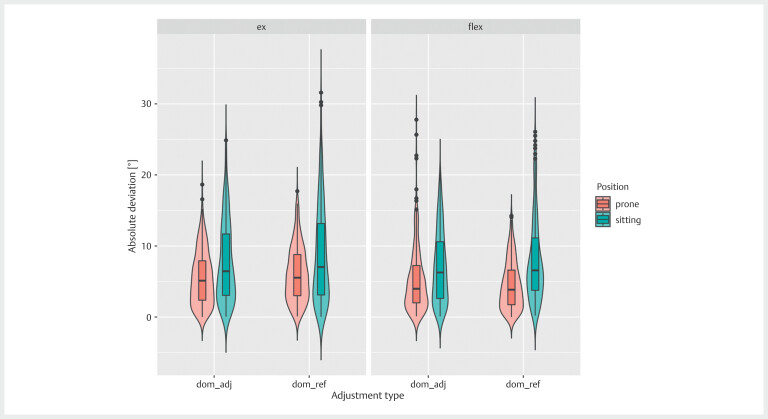
Boxplot of the difference in the knee angle reproduction error between the dominant limb adjusting to or setting the reference angle: dom_adj, the dominant leg was adjusted to replicate the target angle; dom_ref, the dominant leg was set at the reference angle.

Following the identification of significant interaction effects, Tukey’s HSD tests were conducted to further explore the specific differences between the groups. Post hoc testing for the body orientation when controlling for limb dominance revealed a statistically significant difference of *Δ* =3.2° for the body position when the dominant limb was the reference leg ( *t* (1,64)=−5.68, SE=0.56 [95% CI:−4.30,−2.06], *p* < 0.001, *d* =−0.53 [95% CI:−0.83,−0.24]) and when the nondominant limb was the reference leg ( *t* (1,64)=−3.61, SE=0.56 [95% CI:−3.14,−0.904], *p* =0.0006, *d* =0.84 [95% CI:−1.13,−0.54]). A lower AAE resulted when subjects were in the prone position compared to the seated position (dominant limb: *Δ* =−3.2 and nondominant limb: *Δ* =−2.0). In contrast, post hoc testing revealed no statistically significant difference in the prone position between the dominant limb and the nondominant limb for the reference leg ( *t* (1,1977)=0.48, SE=0.23 [95% CI:−0.341, 0.56], *p* =0.63, *d* =−0.03 [95% CI:−0.09, 0.15]). In the sitting position, there was a statistically significant difference between the dominant and nondominant limbs for the reference leg ( *t* (1,1977)=−4.58, SE=0.23 [95% CI:−1.50,−0.60], *p* < 0.001, *d* =−0.28 [95% CI:−0.40,−0.16]) with a 1.1° lower AAE when the nondominant limb served as the reference leg ( Fig. 2 ).

## Discussion

The aim of this study was to investigate the influence of the body orientation, the direction of movement, and limb dominance on the AAE in an active knee angle reproduction test as a measure for proprioceptive acuity. It was hypothesized that there would be significant differences in the AAE when the testing conditions varied. Furthermore, it was assumed that a different AAE would result when the dominant limb was used as the reference leg compared to the nondominant limb. The results indicated that the body orientation and limb dominance were the main factors that influenced the AAE. The reproduction error values were significantly higher in the seated position compared to those in the prone position. The effect sizes ( *d* =0.50–0.80) and differences in the AAE values recorded in the two positions demonstrated that the body position had a clinically important effect, independent of the dominance of the reference leg. Furthermore, the present study showed that the dominance of the reference limb had a significant impact on the resulting AAE in the sitting position but not in the prone position. The AAE was significant when the dominant limb was the reference leg. However, the AAE values differed by only 1° (95% CI: 0.6°, 1.5°), and the effect size was small ( *d* =0.28). According to the recent literature, this small difference between the target and reproduction angles might not be clinically relevant 25 31 .

The present findings are of great practical relevance because numerous studies that have been conducted to evaluate knee proprioception in healthy subjects have lacked validity and comparability due to the use of different testing procedures 1 7 16 32 . Furthermore, no previous study has evaluated the influence of leg dominance on the AAE in combination with different body positions or the direction of the knee movement in a JPS test.

Based on the present results, the ability to reproduce knee angles is seemingly connected to the position applied in the test protocols. This has also been highlighted by other recent studies, which have shown that reproduction errors are significantly different when testing is conducted under different conditions 7 14 15 16 20 . Furthermore, the present results are in line with the recent literature on limb dominance that shows that the results of proprioceptive tests (e.g., return to sports, y-balance, or JPS tests) are dependent on limb dominance 33 34 . Wieber et al. demonstrated that the body orientation and the movement direction influence the resulting AAE in healthy subjects but did not control for limb dominance 7 . Other studies on lateral dominance have shown that there are differences between the dominant and non-dominant legs in an active JPS test 24 35 . A possible reason for the differences in the AAE when the movement direction and body position are varied could be the influence of gravity 36 . The effect of gravity on the hamstrings and quadriceps muscle varies between the sitting and prone positions. In the prone position, gravity-induced torques are greatest on hamstrings, whereas in the seated position they are greatest on the quadriceps muscle 36 . Because the motor unit recruitment differs between the two positions, it is expected that the muscle activity present in the two positions also differs and that the proprioceptive information available in the prone position is different from that available in the seated position 10 37 38 . When moving against gravity, the concentric muscular strain and load on the joint structures is greater 19 . This is also due to the increased activation of the muscle spindles and the Golgi tendon organ during the contraction of the concentric quadriceps muscle 19 .

Furthermore, proprioceptive abilities and the resulting movement patterns are dependent on the tactile feedback 39 . In a seated position, the site of the tactile feedback is primarily the back of the thigh, whereas in a prone position, the tactile feedback sites are more likely to be the front of the thighs and the patellae 40 . Hence, subjects receive different tactile feedbacks when they are in the prone and seated positions. In addition, when a subject is in the prone position, more of their body is in contact with the orthopedic bench, and therefore, they receive additional afferent inputs from cutaneous receptors located in other muscles of the lower limb 13 . These inputs may influence the knee’s proprioceptive input, resulting in a more accurate perception of the knee joint angle in the prone position. Another possible reason for the differences in the AAE recorded in the prone and seated positions could be the difference in the body’s position in space, especially the position of the head (upright in the seated position vs. horizontal in the prone position) 41 .

To date, both positive and negative findings have been obtained regarding limb dominance; therefore, whether limb dominance influences knee proprioception among healthy subjects remains questionable. Most of the available scientific evidence is somewhat limited due to a primary focus on the upper extremities. However, considering that each brain region predominantly controls the limb on the opposite side; this implies that lateralized brain activity is a consistent feature in proprioceptive tasks, regardless of limb dominance 42 43 44 . Multiple investigations conducted to explore brain activity during joint position matching tasks have consistently highlighted a prevalence of right hemisphere activation, irrespective of the active leg 24 35 . However, it is worth noting that conflicting evidence exists: other studies have demonstrated no significant differences between the dominant and nondominant sides 33 45 46 47 48 . Limb dominance has also been shown to be strongly related to physical activity and the extremities that individuals use when they are participating in sports or specific disciplines 33 . Others who have compared the angular reproduction performance of trained and untrained participants have noted variations in both dominant and nondominant limbs 19 21 . Nevertheless, it is difficult to determine the effect of limb dominance in a task with which subjects are unfamiliar 49 . Engagement in training has the potential to positively influence the adaptations of muscle spindles and induce changes in the central nervous system, including the enhancement of synaptic connections 21 . Within the scope of the present study, the subjects exhibited physical activity, as evidenced by the Tegner activity scale 50 .

### Limitations

In the present study, no correlation analysis was conducted between physical activity, types of sports (e.g., predominantly upper body or lower body involved) or the training load and JPS accuracy. The potential causal relationship between the type and the training load/intensity of sports or physical activity and the JPS could be a point for discussion. This aspect remains an intriguing area for further investigation. A further limitation is that the JPS test used may not fully capture comprehensive sensorimotor performance during functional tasks involving multi joint movements in a weight-bearing setting. The methodology adopted for measuring the articular range of motion does not ensure the minimization of measurement errors deriving from the potential three-dimensional motion of the joints. We intentionally selected the utilized methodology due to its similarity to the return-to-sports tests used after knee injuries, which are mostly conducted in a non-weight bearing setting and without cost-intensive equipment 25 31 . Therapeutic assessment involving weight-bearing tasks may not always be feasible, particularly during the acute phase of injury 33 . It is conceivable that a more challenging task could have elicited differences in the resulting AAE, but the results of this study therefore provide higher generalizability to the JPS measurement of patients during rehabilitation. The AAE was the main outcome measurement parameter assessed. Further parameters, for example the variable error, still provide an indication of the accuracy of the measurement but were not included as the AAE has proven to be a more reliable parameter in the recent literature on the JPS 15 16 . Furthermore, a causal relationship can be generalized to different times, but lower generalizability for different users due to the standardized complex application of the device. Furthermore, the subjects had to perform the JPS test at a self-selected pace. It is worth noting that movement velocity may influence neuromuscular activity and thus potentially affect the resulting AAE 33 37 .

Future studies could be conducted on possible deviations in the knee angle reproduction error between injured and non-injured sides with regard to the limb dominance and what influence a particular type of sport may have on limb dominance and proprioception in the knee.

## Conclusions

The aim of this study was to investigate the influences of body orientation, direction of movement, and limb dominance on the knee angle reproduction error in an active JPS test as a measure of proprioceptive acuity. The body orientation was found to greatly influence the knee angle reproduction error, showing that reproduction failure is greater in a sitting position than in a prone position, especially if the dominant limb is chosen as the reference leg. Practitioners are advised to use standardized test procedures to progress their clinical value and implement in daily practice. The results obtained in different settings should be critically analyzed; directly comparing the results obtained in the prone and sitting positions is not recommended. However, the dominance of the reference limb might be relevant when testing patients and comparing healthy and injured knees. Future studies should focus on possible differences in the knee angle reproduction error between the injured and non-injured sides with regard to limb dominance.
